# A case report of anastomosing hemangioma of the ovary

**DOI:** 10.1097/MD.0000000000033801

**Published:** 2023-05-12

**Authors:** Zhen Wang, Junbo Hu

**Affiliations:** a Hubei University of Medicine, Shiyan, China; b Department of Pathology, Maternal and Child Health Hospital of Hubei Province, Wuhan, China.

**Keywords:** anastomosing hemangioma, clinicopathological features, diagnosis, ovary

## Abstract

Anastomosing hemangioma (AH) is a rare benign neoplastic vascular lesion that histologically resembles a well-differentiated angiosarcoma. AH commonly involves the urinary system and testes. However, these tumors can also involve the ovaries in some rare cases. This manuscript presents the case of a 28-year-old Chinese woman diagnosed with ovarian AH.

**Patient concerns:**

The woman was admitted to the hospital with a 4-month history of a right ovarian mass discovered by ultrasound (US) after a spontaneous abortion. The US examination showed a 4 cm × 4 cm irregularly shaped mass with a rich blood supply.

**Diagnoses:**

AH of the right ovar.

**Intervention:**

The patient underwent laparoscopic surgery to remove the mass. The postoperative pathological examination revealed that the mass contained capillaries arranged in a characteristic anastomotic or confluent pattern commonly seen in AHs.

**Outcomes:**

The mass was successfully removed. The follow-up examination at 7 months post-surgery showed that the patient recovered well, and no recurrence or metastasis was found.

**Lessons:**

AH of the ovary is a rare benign vascular tumor. On imaging examinations, AHs appear as mostly well-defined, heterogeneous nodules with peripheral enhancement as other benign nodules. However, a definitive diagnosis can only be achieved through histopathological examination.

## 1. Introduction

Anastomosing hemangioma (AH) is a rare benign neoplastic vascular lesion that histologically resembles well-differentiated angiosarcoma. AH typically involves the testes, the urinary systems,^[1]^ and occasionally the liver,^[2]^ gastrointestinal tract,^[3]^ and paraspinal region.^[4]^ However, AH can also affect the ovaries in some rare cases. In this report, we present the case of a woman diagnosed with ovarian AH. In this case study, we have retrospectively analyzed the clinicopathological features, imaging findings, immunophenotype, and differential diagnosis of this tumor. In addition, we have compared this case with other ovarian AH cases found in the literature.

## 2. Case report

**Figure 1.**
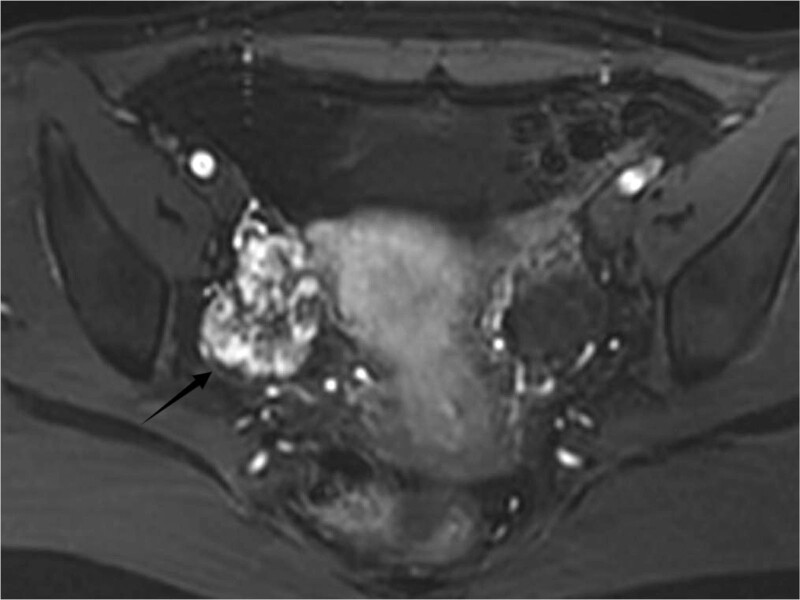
Magnetic resonance T1WI showed a space-occupying lesion in the right adnexal area.

A 28-year-old woman was admitted to our hospital after a spontaneous abortion, whereby an ultrasound (US) revealed a right ovarian mass that had been present for 4 months. The woman had a total of 3 pregnancies and 3 miscarriages. The US examination revealed a 4.0 cm × 4.0 cm irregular mass occupying the parenchyma of the right ovary, with an abundant blood supply. The magnetic resonance imaging examination revealed an abnormal circular mass measuring 4.1 cm × 4.0 cm × 3.6 cm within the adnexal area that occupied most of the ovary. The mass displayed a slightly non-uniform hyperintense signal on both T1 and T2-weighted images (Fig.1). The serum blood examination revealed a normal cancer antigen 125 level.

A laparoscopic ovarian cystectomy was performed to remove the mass. During the surgical procedure, significant adhesions were noted between the right ovary and the posterior leaf of the right broad ligament. The removal of the adhesions revealed an enlarged ovary. A yellowish-white solid mass with an irregular vascular surface measuring about 4.0 cm × 4.0 cm × 4.0 cm was seen invading the ovarian cortex.

**Figure 2.**
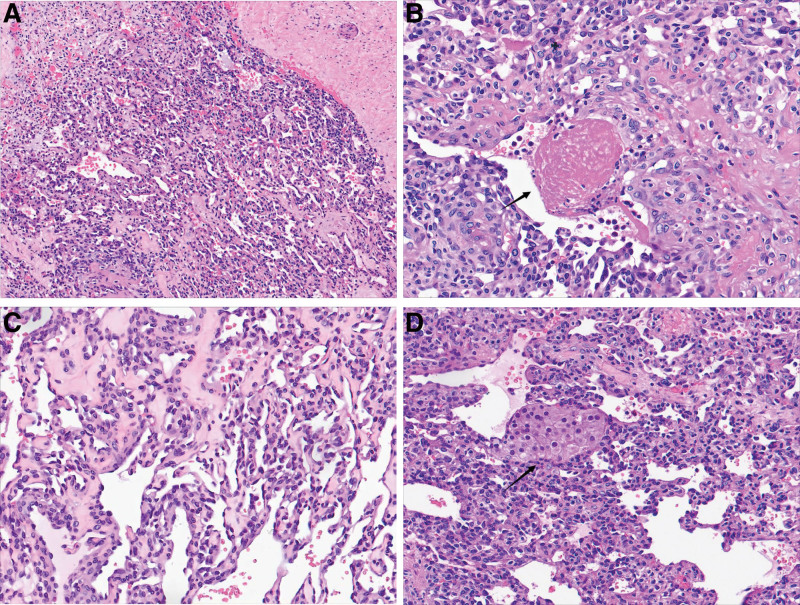
Microscopic examination of the mass. (A) tumor showing a well-defined boundary with capillaries distributed in an anastomotic pattern, forming sinusoid-like lumens with red blood cells. The tumor also displayed anastomotic sinus-like structures (hematoxylin and eosin [HE], magnification, ×40). (B) Fibrin thrombi (HE × 100). (C) endothelial cells show no or mild nuclear variability and scattered hobnail cells (HE × 400). (D) Leydig cells surrounding the hemangioma (HE × 200).

**Figure 3.**
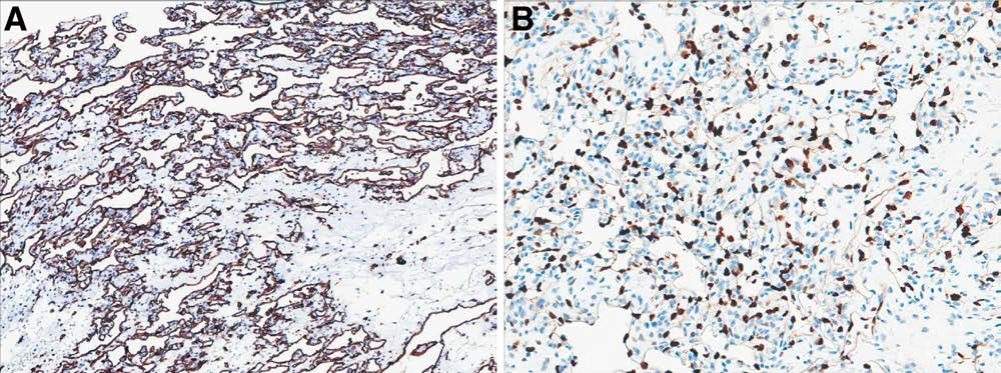
(A) Diffuse and strong expression of CD31 in the inner vascular layer (CD31, ×100). (B) ERG staining in the cells lining the vessels (ERG, ×100).

The gross pathological examination revealed a gray-red broken cyst wall surrounding the mass and a yellowish-white irregular nodular tissue measuring 2.5 cm × 2.0 cm × 0.8 cm inside the mass. The microscopic examination showed a multi-nodular growth with relatively regular borders (Fig. 2A). Under low or medium magnification, the capillaries in the sinusoid lumen appeared to have an anastomosis or traffic-like pattern. Some loose areas with edema were noted together with a fibrin thrombus (Fig. 2B). Under high magnification, the tumor appeared as a plump single layer of endothelial cells. The nuclei of the cells protruded into the cavity and displayed a “shoe-nail” appearance (Fig. 2C). No other significant abnormal features, such as atypia or mitotic figures, were identified in the nuclei. Leydig cells were also observed around the hemangioma (Fig. 2D). A fibrous stroma separated the vascular tissue in most areas, and adipose tissue was also observed in some areas. The results of the immunophenotype analysis showed that the tumor cells expressed the CD31 (Fig. 3A), CD34, FLi1, ERG (Fig. 3B), and SF-1 proteins and partially expressed cyclin D1. However, the tumor cells did not express D2-40, CK5/6, PAX8, desmin, inhibin, calretinin, CD99, WT1, CD10, catenin-β, EMA, or CK7. The Kiel-67 (Ki67) proliferation index was approximately 5%. Based on the histopathological findings, the patient was diagnosed with an AH of the right ovary. Genetic analysis revealed no abnormalities in the expression of GNAQ (p.Q209H) and GNA14 (p.Q205L) genes.

The patient was reviewed after 7 months. The follow-up examinations showed that the patient recovered well after the operation, and the imaging examinations revealed no evidence of recurrence or metastasis.

## 3. Discussion

AH is a rare benign neoplastic vascular lesion named for its typical irregular, anastomotic sinusoid vascular lumen structure. This disease was first described by Montgomery et al in 2009.^[5]^ Although AH can originate from various sites, ovarian AH is especially rare. To date, only about 30 cases have been reported in the literature published in English, and only 1 case has been reported in the literature published in Chinese.^[6]^ Through this literature review, we found that most cases occurred between 43 and 81 years. However, in this case study, we report on a rare case of ovarian AH in a 28-year-old Chinese woman. To our knowledge, this is the youngest patient diagnosed with ovarian AH.

Based on our literature review, we found that the maximum diameter of the ovarian AH tumors ranged from 0.1 cm to 11.2 cm. Most cases affected a single ovary and were diagnosed accidentally. Some patients presented with adnexal masses or benign serous cystadenomas. In this case, the adnexal mass was found accidentally during a follow-up US performed after a miscarriage. Ovarian AH shows the typical features of benign lesions on US examinations, which include regular borders and uneven density, with peripheral enhancement. However, in a small number of cases, ovarian AH can be misdiagnosed as a malignant epithelial tumor or angiosarcoma due to the presence of ascites and elevated cancer antigen 125.^[7,8]^ Therefore a histopathological assessment is necessary to obtain a definitive diagnosis.

Histopathological analysis of ovarian AH typically reveals focal anastomosis, communication between capillaries, and lobulated hyperplasia. This arrangement resembles the sinusoidal architecture of the red pulp in the spleen. The AH tissue shows edematous and hyaline stroma with hobnail-like endothelial cells arranged within it, and lymphocyte infiltration may also be present. The endothelial cells appear mildly abnormal, with no evident stratification, cellular atypia, or mitotic figures. In some cases, an intracapillary fibrin thrombus is also observed.^[7,9]^ AH cases with adipocyte metaplasia^[10]^ and extramedullary hematopoiesis have also been reported.^[11]^ According to the fifth edition of the World Health Organization classification of soft tissue tumors, the diagnosis of AH requires the presence of specific criteria, including the presence of hobnail-shaped endothelial cells that line the vascular lumen and form anastomoses with each other, hyaline droplets and extramedullary hematopoiesis.^[12]^ In this case, the microscopic examination of the tumor fully met the World Health Organization diagnostic criteria. In addition, because the hilar cells are special mesenchymal cells located at the hilum of the ovary, they have a characteristic cytoplasm that is rich in cholesterol and lipochrome. Furthermore, Reinke crystals are also characteristic features of AH. However, these crystals may not always be present, and their absence does not rule out a diagnosis of AH.^[9,11,13]^ Other common histological features reported in most of the literature reports include (21/31) significant interstitial luteinization and eosinophilic or vacuolated cytoplasm. These features can be easily confused with other types of ovarian tumors, such as steroid cell tumors, Leydig cell tumors, or other mixed stromal vascular tumors.

It has been suggested that the high estrogen levels produced by interstitial luteinization can stimulate the growth of hemangiomas.^[14]^ Another theory proposes that the growth of hemangiomas can lead to the compression of ovarian parenchyma, which may result in the development of luteinized cells.^[15]^ However, the relationship between hemangiomas and luteinization is not yet fully understood, and there is currently no clear evidence to support this hypothesis.

Studies have shown that AH cells can diffusely express vascular endothelial immunohistochemical markers, such as EGR, FLi1, CD31, CD34, and FⅧ. On the other hand, CD31 and CD34 are expressed in almost all cases. These findings suggest that the cells in ovarian AH may have either vascular endothelial differentiation or a neoplastic origin related to the vasculature, Consistent with our case. CK, D2-40, Desmin, α-Inhibin, and other markers are not generally expressed in ovarian AH. Additionally, ovarian AH typically exhibits a low Ki67 index, which indicates a low tumor proliferation rate.

The genetic analysis of AH patients worldwide showed that GNAQ(p.Q209H) mutations had been found in several AH cases originating from various sites but not the ovaries. Only 1 case of GNA14 p.Q205L hotspot mutation was found in ovarian AH.^[16,17]^ However, none of these mutations were found in our case study. There are 2 potential reasons why these mutations are not always observed in ovarian AH cases. First, the rarity of this type of ovarian tumor limits the number of cases available for analysis. In addition, the occurrence of these mutations may vary in different sites. Therefore, further research and analysis are necessary to determine the significance of genetic mutations in the pathogenesis of AH.

Differential diagnosis of ovarian AH from other similar tumors, including angiosarcoma, epithelioid hemangioma, yolk cystoma, and sex cord-stromal tumors, is essential to treat ovarian tumors effectively. Similar to AH, well-differentiated angiosarcoma can also express EGR, CD31, CD34, and other vascular endothelial immunohistochemical markers, and they also present with anastomosed vascular channels. However, angiosarcomas have some distinct features, which include infiltrative lesions marked by nuclear atypia, high mitotic activity, multilayered endothelial cells, and substantial hyperplasia and necrosis. Epithelioid hemangiomas are mainly composed of well-formed vessels. These tumors do not show the traffic or anastomosis-like cracks, and the vascular endothelial cells with abundant eosinophilic cytoplasm typically found in AH. Yolk cystoma occurs in young women and tends to present with highly elevated serum alpha-fetoprotein. Compared with AH, the tissue structure of yolk cystoma tends to be diverse, highly heterogeneous, and mitotically active. The tissue can also differentiate into a variety of endodermal structures. Patients with sex cord-stromal tumors tend to present with irregular vaginal bleeding and menstrual disorders. In addition, immunohistochemical analysis revealed a diffused expression of EGR, FLi1, CD31, CD34, and other vascular endothelial immunohistochemical markers and a negative expression of CK, D2-40, Desmin, α-Inhibin, and EMA could be used to rule out a diagnosis of sex cord-stromal tumors.

## 4. Conclusions

In summary, ovarian AH is a rare benign vascular lesion with a good prognosis. However, due to its clinical manifestation, ovarian AH can be easily misdiagnosed as ovarian angiosarcoma or other malignant epithelial tumors. The findings of this case study have improved the understanding of ovarian AH and could be used by clinicians to facilitate the differential diagnosis of ovarian AH and avoid overtreatment.

## Acknowledgments

The authors express their gratitude to the patient who made this work possible, as well as the professionals and researchers that participated in this study. A patient informed consent was acquired.

## Author contributions

**Conceptualization:** Junbo Hu.

Data curation: Zhen Wang.

Formal analysis: Zhen Wang, Junbo Hu.

Investigation: Zhen Wang, Junbo Hu.

Resources: Zhen Wang, Junbo Hu.

Writing – original draft: Zhen Wang.

Writing – review & editing: Zhen Wang, Junbo Hu.
